# Are we creating a new phenotype? Physiological barriers and ethical considerations in the treatment of hereditary transthyretin-amyloidosis

**DOI:** 10.1186/s42466-021-00155-8

**Published:** 2021-11-01

**Authors:** Maike F. Dohrn, Jessica Medina, Karmele R. Olaciregui Dague, Ernst Hund

**Affiliations:** 1 Department of Neurology, Medical Faculty of the RWTH Aachen University, Neuromuscular Outpatient Clinic, University Hospital Aachen, Pauwelsstr. 30, 52074 Aachen, Germany; 2 Dr. John T. Macdonald Foundation, Department of Human Genetics and John P. Hussman Institute for Human Genomics, University of Miami, Miller School of Medicine, Miami, FL USA; 3 Department of Epileptology, Medical Faculty, University Hospital Bonn, Bonn, Germany; 4 Amyloidosis Center Heidelberg, Heidelberg University Hospital, Heidelberg, Germany; 5 Department of Neurology, Heidelberg University Hospital, Heidelberg, Germany

**Keywords:** Central nervous system amyloidosis, Eye involvement, Blood–brain barrier, Liver transplantation, Antisense oligonucleotides, Small interfering RNA, Intrathecal administration, Allele-specific genetic therapy, CRISPR

## Abstract

Hereditary transthyretin (TTR) amyloidosis (ATTRv) is an autosomal dominant, systemic disease transmitted by amyloidogenic mutations in the *TTR* gene. To prevent the otherwise fatal disease course, TTR stabilizers and mRNA silencing antisense drugs are currently approved treatment options. With 90% of the amyloidogenic protein produced by the liver, disease progression including polyneuropathy and cardiomyopathy, the two most prominent manifestations, can successfully be halted by hepatic drug targeting or—formerly—liver transplantation. Certain *TTR* variants, however, favor disease manifestations in the central nervous system (CNS) or eyes, which is mostly associated with TTR production in the choroid plexus and retina. These compartments cannot be sufficiently reached by any of the approved medications. From liver-transplanted patients, we have learned that with longer lifespans, such CNS manifestations become more relevant over time, even if the underlying *TTR* mutation is not primarily associated with such. Are we therefore creating a new phenotype? Prolonging life will most likely lead to a shift in the phenotypic spectrum, enabling manifestations like blindness, dementia, and cerebral hemorrhage to come out of the disease background. To overcome the first therapeutic limitation, the blood–brain barrier, we might be able to learn from other antisense drugs currently being used in research or even being approved for primary neurodegenerative CNS diseases like spinal muscular atrophy or Alzheimer’s disease. But what effects will unselective CNS TTR knock-down have considering its role in neuroprotection? A potential approach to overcome this second limitiation might be allele-specific targeting, which is, however, still far from clinical trials. Ethical standpoints underline the need for seamless data collection to enable more evidence-based decisions and for thoughtful consenting in research and clinical practice. We conclude that the current advances in treating ATTRv amyloidosis have become a meaningful example for mechanism-based treatment. With its great success in improving patient life spans, we will still have to face new challenges including shifts in the phenotype spectrum and the ongoing need for improved treatment precision. Further investigation is needed to address these closed barriers and open questions.

## Background

Transthyretin (TTR) amyloidosis is a systemic disease caused by dissociation of the TTR tetramer and subsequent fibril deposition in tissues [1]. Despite the existence of a less aggressive disease form associated with wild-type TTR [2], this mechanism is attributed to more than 130 known amyloidogenic missense mutations in the *TTR* gene (OMIM *176300). With an autosomal dominant mode of inheritance, hereditary TTR (ATTRv) amyloidosis typically becomes symptomatic with a progressive sensorimotor and autonomic neuropathy and cardiac dysfunction [1]. Rarer manifestations include proteinuria, vitreous opacities, cerebral hemorrhage, stroke-like episodes, and dementia [3, 4]. It is so far unknown, why ATTR amyloid preferentially deposits in certain organs but not in others. Manifestation subtypes can loosely be correlated with the respective underlying *TTR* variant; however, this is not the only factor of influence. While the genetic cause of ATTRv amyloidosis itself is well understood and even targetable by several treatment options, important aspects like differences in organ tropism and manifestation onset are not well understood to date. As potential modifiers, influencing factors in the extracellular matrix, in the *TTR* gene itself or in other genes like *RBP4* [5] have been discussed in the literature (for review see [6]). It further seems to play a role whether a patient or the affected antecedent is male or female, suggesting that X-linked genes like *AR* affect TTR amyloidogenesis as well [7]. Furthermore, borderline CAG repeat expansions in the *ATXN2* gene are associated with an earlier age of onset [8].

The most prominent physiological function of TTR is transporting thyroxin and retinol binding protein in serum and cerebrospinal fluid (CSF) [9–11]. Whereas this function can sufficiently be compensated by other proteins such as thyroglobulin or albumin in serum, TTR is the main thyroxin transporter in the CNS [12]. Due to its proteolytic function and interaction with Aß proteins, a protective role in Alzheimer’s disease has been previously discussed [13] (for review see [14]).

**Fig. 1:**
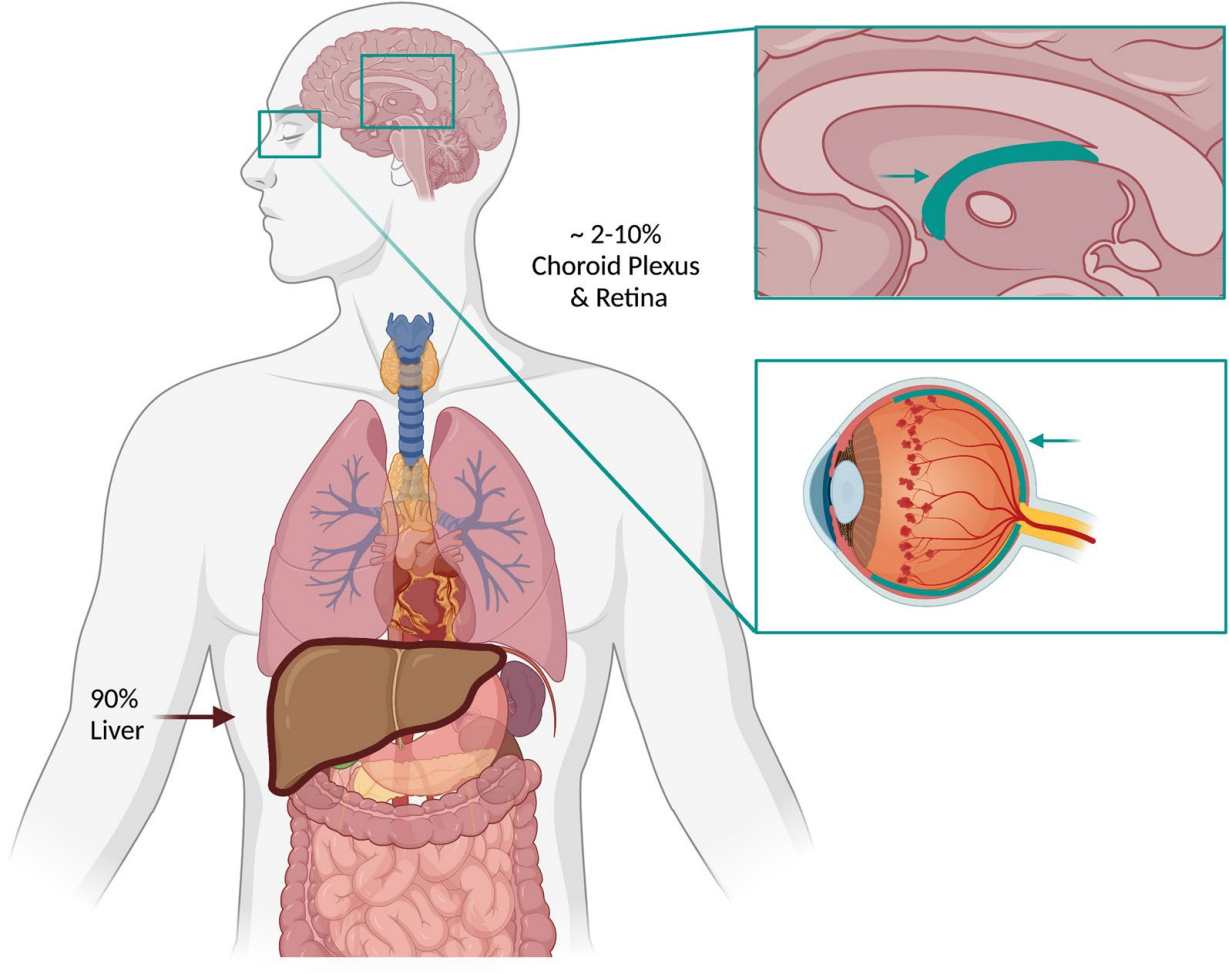
Physiological TTR production sites. Whereas 90% of TTR is of hepatic origin, other production sites are the choroid plexus and the retina. This becomes of growing importance as these two compartments cannot be sufficiently targeted by currently approved medications. Created with BioRender.com

Considering that roughly 90% of TTR is hepatically produced (Fig. 1), liver transplants function similarly to gene therapy. With great success prolonging both span and quality of life, the first liver transplantations were performed in the early 1990s [15–17]. Worldwide, 2,181 patients have been registered so far as organ recipients due to ATTRv amyloidosis (http://www.fapwtr.org/ram_fap.htm). Other than surgically, hepatic TTR production can now be targeted by two approved mRNA silencing drugs: patisiran is a small interfering RNA molecule [18] and inotersen an antisense oligonucleotide [19] that both lower serum TTR levels up to 20% of normal, resulting in significant improvement of neuropathy and cardiomyopathy compared to placebo-treated controls. Other than reducing TTR production, the approved substance tafamidis [20, 21], available in daily oral dosages of 20 mg and 61 mg, has been shown to stabilize the TTR tetramer and thereby to decelerate the clinical course including neuropathy and cardiomyopathy, improving overall survival without major side effects.

If treated appropriately, ATTRv amyloidosis might therefore no longer be fatal. This might confront patients and caregivers with new challenges in the future. Out of the currently approved treatments, tafamidis is the only one that crosses the blood–brain barrier, but only minimally [22]. Previous studies have shown that CSF TTR is mainly produced in the choroid plexus [12], and can thereby not be influenced by hepatic TTR mRNA degradation. On the other hand, TTR is least replaceable in the CNS, with little known proteins able to take over its function. This places unique challenges on overall TTR transcript knockdown approaches. Recognizing its neuroprotective role in Alzheimer’s disease [23], it seems paradox that TTR can both prevent and cause dementia.

In this review, we will critically discuss the role of TTR amyloidogenesis in the CNS as well as the subsequent therapeutic challenges. We will summarize the current literature, point towards open questions, and implement an ethical discussion of potential therapeutic long-term effects.

## Review

### Non-liver-derived transthyretin and associated disease manifestations

**Fig. 2:**
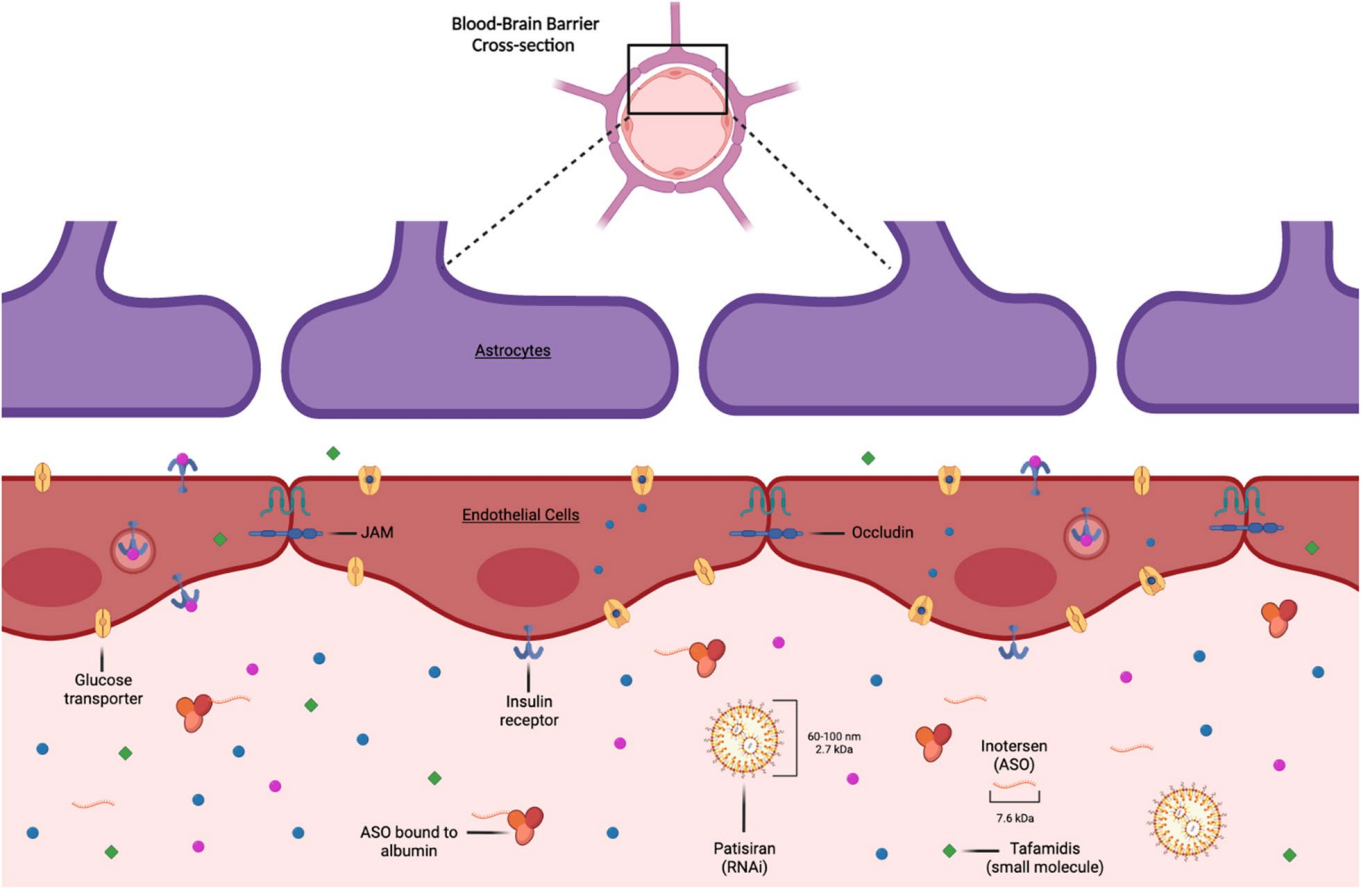
Blood–brain barrier. Schematic depiction of the anatomy and function of the blood–brain barrier. ASO, antisence oligonucleotide; JAM, junctional adhesion molecule; RNAi, ribonucleotide acid interference drug. Created with BioRender.com

To an extent of about 10% [12], TTR is not produced by the liver, but by the choroid plexus and the retinal epithelium (Fig. 1). Protected by the blood–brain barrier (Fig. 2), these central compartments of TTR production are not accessible by large molecules or antisense drugs, neither are they influenced by the effects of liver transplant.

Clinically, ocular ATTR deposits cause vitreous or lens opacities, chronic open-angle or neovascular glaucoma, keratoconjunctivits sicca, abnormalities of conjunctival or retinal blood vessels, or optic neuropathy [24, 25]. The clinical spectrum of CNS manifestations is broad, comprising radiculopathy, subarachnoid and intraparenchymal haemorrhage, stroke-like episodes that might as well be explained by seizures, and periods of decreased consciousness [3, 26–28].

In the natural course of ATTRv amyloidosis, several variants, including the most frequent, “Portuguese”, mutation p.Val50Met, have CNS or ocular manifestations in their phenotypic spectrum [24]. A predominant eye involvement has been described for the variants p.Arg54Gly, p.Lys55Thr, p.Trp61Leu, p.Tyr89His, and p.Gly83Arg. Leading CNS manifestations have been associated with the variants p.Leu33Pro, p.Asp38Gly, p.Ala45Thr, p.Val50Cys, p.Tyr69Pro, p.Tyr89His, and p.Tyr134Cys. It is interesting to observe that these variants are not the same. It is not understood, however, why one variant increases the liability to one specific, but not to other organ manifestations.

Following liver transplant, Ando et al. described ongoing or new vitreous and leptomeningeal disease manifestations [25]. These were not attributed to wild-type, but to variant TTR, which means that in contrast to heart and other peripheral tissues, these deposits were not formed around previously seeded mutant-derived amyloid, but that there was still new disease activity even after liver transplantation.

Serum TTR is in fact relatively under-represented in the vitreous bodies and CSF, but it is mainly produced by the retina and choroid plexus. In accordance with these observations, patients with iatrogenic ATTRv amyloidosis, meaning recipients of domino-transplanted, fully functioning organs carrying an amyloidogenic *TTR* mutation, have so far not been observed to develop symptoms other than neuropathy and cardiomyopathy [29–32], which both result from the presence of unstable TTR in the peripheral circulation. Accordingly, ATTR_wt_ amyloidosis has so far not been described in association with CNS and eye manifestations in the literature.

All three drugs so far approved for the treatment of ATTR amyloidosis (Table 1) have a very limited (tafamidis) or no (patisiran, inotersen) capacity to cross the blood–brain barrier [22]. What we have learned from liver transplanted patients might therefore be applicable in the context of long-term treatment with any of these drugs. Even though CNS and eye manifestations are not the leading phenotype for the majority of *TTR* variants, longer lifespans enable a different phenotypic pattern under treatment.

**Tabel 1:** Basic information and pharmakokinetics

	Tafamidis (Vyndaqel™/Vyndamax™)	Patisiran (Onpattro™)	Vutrisiran^a^	Inotersen (Tegsedi™)	Eplontersen^a^	TTR
Dosage	20/61 mg	0.3 mg per kg body weight	25 mg	300 mg	45 mg	NA
Application	oral	i.v.	s.c.	s.c.	s.c.	NA
Frequency	1×/d	1×/3 weeks	1×/3 months	1×/week	1×/4 weeks	NA
Effect site	Liver/blood	Liver	Liver	Liver	Liver	NA
Uptake mechanism/receptors	Unspecific	Unspecific	Specific trough GalNaC modification via ASGPR	Unspecific	Specific trough GalNaC modification via ASGPR	NA
Molecule size [D]	308.12	14,304	MD	7600.8	9046.1	55 K, 16 K g/mol
Plasma t_1/2_	24 h	0.79 h	4.8 h	3.9 h	1.6 h	48 h
Tissue distribution	Liver and plasma	Liver ≫ lymphatic tissues > kidneys, lungs, heart, adrenals	Liver, kidneys, injection site	Liver, kidney, lymphatic tissues, injection site, bone marrow	Liver, kidney, injection site	Plasma, CSF, eyes, liver, kidney, pancreas
BBB passage	Partial (1.5%)	None	None	None	None	2–10% synthesis in choroid plexus and retina

Synoptic summary on the three approved drugs tafamidis, patisiran, and inotersen, and the two drugs vutrisiran and eplontersen that are currently in phase III trials. Data were provided by the respective pharma companies (Pfizer, Alnylam, and Ionis). If the exact information was not being publicly available at the time when this paper was written (09/2021) or provided upon request, we indicated this with MD (missing data). For comparison, we provide similar information on the TTR protein on the right. ASGPR, asialoglycoprotein receptor receptor; BBB, blood–brain barrier; CSF, cerebrospinal fluid; GalNac, NA, N-Acetylgalactosamine; h, hour(s); i.v., intraveneous; NA, not applicable; s.c., subcutaneous; t_1/2_, half-life

^a^Drug not (yet) approved in Europe or in the United States of America

### The blood–brain barrier and its therapeutic implications

The blood–brain barrier (Fig. 2) consists of non-fenestrated endothelial cells that are closely connected by tight junctions and surrounded by astrocytes and pericytes. Several factors limit the permeability of a systematically delivered therapeutic into the CNS, but none more restrictively than size (less than 4 nm) and charge (preferably uncharged) (reviewed further in [33]). Following these two rules, most therapeutic molecules, including RNA/DNA-based approaches, are both too big and too polar to cross.

The evolution of transient genetic medicine, like RNA interference (RNAi) and antisense oligonucleotides (ASOs) requires either a carrier delivery system or modified RNA or DNA backbone chemistry to increase circulation and half-life [34, 35]. Unmodified RNA or DNA sequences used in RNAi or ASOs tend to quickly exit circulation through renal filtration and limit their intracellular survival against endo- and exo-nucleases [34, 36]. Bearing this in mind, it is patently clear why the most efficacious molecular adaptations made to these strategies are counterproductive for their systemic and targeted distribution. For this reason, the most obvious solution is direct administration into the CNS via intrathecal delivery, a method adapted for the treatment of aggressively progressive disorders like spinal muscular atrophy (SMA), amyotrophic lateral sclerosis (ALS), and Huntington’s Disease [37]. Currently, nusinersen is the only intrathecally delivered ASO approved by the FDA and EMA [38, 39], however, several others are in trial for the previously mentioned disorders.

In ATTRv amyloidosis, the small molecule tafamidis is the only approved therapeutic that can cross the blood–brain barrier, however, no more than 1.5% of the plasma circulating drug actually reach the cerebrospinal fluid [22]. Like tafamidis, the catechol-O-methyltransferase (COMT) inhibitor tolcapone, approved for the treatment of Parkinson’s disease, has a TTR stabilizing effect [40] with a greater capacity to cross the blood–brain barrier [41, 42]. Relevant limitations of this drug are, however, the short half-life and the potential side effects including liver failure.

Using Watson–Crick base pairing, antisense oligonucleotide and RNA-based strategies have historically elicited knockdown mechanisms as plausible treatment approaches, especially for dominantly inherited disorders. This is the case for both patisiran, a double stranded RNA-interference (RNAi) therapeutic, and inotersen, a single-stranded antisense oligonucleotide [43, 44]. While the mechanism of action differs, both aim to indiscriminately reduce wildtype and mutant TTR RNA transcripts by targeting a 3’-UTR sequence in the pre-mRNA, ultimately reducing aggregate formation.

Patisiran uses the well-established siRNA strategy of binding to a target RNA transcript and recruiting the RNA-induced silencing complex (RISC) to cleave the transcript, exposing it to exonucleases, subsequent degradation and eventual clearance, but allowing the siRNA to continue binding to other target transcripts. Packaged into a lipid nanoparticle delivery system targeted to the liver, it has proven efficacious in knocking down TTR transcripts in the most relevant organ for TTR production and remaining active for weeks with mitigated secondary and no documented off-target effects. However, it fails to address the ongoing need for targeting production of TTR in the choroid plexus, where continued transcription of amyloidogenic mutant alleles progressively endanger susceptible tissues, including CNS and eyes. Direct and titrated administration of patisiran into the CNS has yet to be published, potentially due to the perceived negligible advantages to repeated intrathecal delivery to tissue not primarily responsible for the majority of TTR production. Underlining this logic is the expressed improvement of patients in regards to their neuropathy and cardiomyopathy [18, 45, 46], both not requiring the drug to cross the blood–brain barrier. However, an intrathecal route of administration is currently under investigation for siRNA therapeutics targeting neurodegenerative diseases like Alzheimer’s or Huntington’s disease. For these indications, phase 1 clinical trials are expected to start recruiting soon (information provided by Alnylam, manufacturer of patisiran).

Inotersen uses a short 20 bp chemically modified DNA sequence to bind a complimentary section of the TTR pre-mRNA 3′-UTR, leading to the recruitment of RNAse H1, a nuclear ribonuclease recognizing DNA-RNA duplexes, and cleavage of the target transcript. Similarly to patisiran, cleavage and clearance of the target transcript permit the ASO to effectively reduce TTR translation. The ASO half-life and resistance to intracellular degradation is owed to its charged backbone and 2’-O-methoxyethyl substitutions in the ribose sugar as well as the phosphorothioate substitutions to the oxygen in the phosphate group. These modifications confer both stability and affinity to a target transcript but impede its penetrance to the CNS [47]. As mentioned above, nusinersen, another ASO drug, has already been approved for intrathecal treatment of spinal muscular atrophy [38, 39]. The mechanism used here is not RNA degradation, but expression regulation through splice modifications. However, the fact that another drug of the ASO family has already been shown to be safely administered to the CSF gives hope for future developments in other indications.

### TTR knockdown in CNS: potential limitations related to TTR-function

If the route of administration was no longer a limitation for CNS TTR knockdown, would it then be its irreplaceable function?

In the CSF, TTR represents 20% of total proteins, with levels ranging between 1.5 and 2.5 mg/dL [48]. Besides the choroid plexus, neurons themselves might contribute to the central TTR production, which has been discussed as a response to neuronal stress. The amount of this effect has not been quantified to date. Several animal studies suggested a neuroprotective role of TTR in the context of peripheral nerve injury [49] or cerebral ischemia [50]. TTR stability seemed to be a positive predictor for favorable outcome in a large screening study on strokes in young adults [51]. In several studies on Alzheimer’s disease (AD), CSF TTR levels were shown to be reduced [52, 53], however, this does not seem to be specific for AD [54] nor consistently reproduced by other studies [55]. How exactly TTR interacts with Aβ and attenuates its toxicity, is not fully understood to date (for review see [14]). The proteolytic cleavage of Aβ fibrils by TTR [56] is one of the hypothesized mechanisms. As shown in cell culture, the uptake of Aβ protein correlated with TTR stability [44]. The overexpression of TTR improved cognition and reduced the neuropathological disease load in mice [57], whereas TTR knock-out led to an accelerated disease course [57]. Beyond AD disease models, TTR knockdown seemed to result in reduced memory function in otherwise healthy mice [58]. Other discussed neuroprotective roles of TTR comprise neuro-regeneration and neuropeptide homeostasis [9].

While some of these TTR functions are closely related to its role in transporting retinol binding protein [58], others might require a direct interaction between TTR and other proteins. These considerations might become important in a (iatrogenic) loss-of-function situation. While the former could be partially replaced by vitamin A substitution, which is already an established requirement under antisense drug medication [18, 19], the latter needs to be further investigated in order to fully assess and address potential risks.

### Potential answers/strategies

In summary, we encounter two major challenges when trying to treat CNS ATTRv amyloidosis:

1. How to target the CNS with the lowest possible toxicity and application burden for the patient?
2. How to selectively reduce the expression of the mutant, but not of the essential wildtype allele?

Therefore, the next intuitive frontier of genetic medicine is both optimizing tissue specific distribution and molecular precision.

Increasing tissue specificity is an important step to reduce overall toxicity. For the indication ATTRv amyloidosis, several translation modifying drugs (vutrisiran, eplontersen) are currently in phase 3 clinical trials that due to a modification with *N*-acetylgalactosamine (GalNAc) have an enhanced delivery to the liver [59, 60]. Unfortunately, there is no such selective, receptor mediated uptake system for the CNS that could be exploited to date.

Targeted delivery of intrathecally administered therapeutics has just recently begun to play a role in ATTRv amyloidosis: Alnylam Pharmaceuticals has conducted pre-clinical studies, using murine models for targeted ocular TTR transcript knockdown (Nair_OTS2018 (alnylam.com)). Achieving sustained circulation through CSF and increased tissue specific penetrance overcomes the most challenging barriers in CNS specific delivery.

Targeting allele-specific RNA transcripts comes with a unique sequence and gene dependent challenge as distinguishing between the two transcripts depends on how specifically the complimentary therapeutic can bind to one, but not the other allele. For the purpose of CNS-delivered genetic therapeutics, an allele- specific approach would permit for wt TTR to remain the primary thyroxin and retinol binding protein transporter, while significantly diminishing the mutant transcripts. Molecular precision can be thought as an interplay between sequence specificity for optimal siRNA or ASO binding and increased therapeutic half-life for continued transcript clearance with mitigated intracellular toxicity. Calibrating appropriate therapeutic dosing is further complicated by the variability in DNA/RNA backbone modifications made to the therapeutic itself. Most FDA-approved DNA/RNA therapies have a handful of tried-and-true substitutions conferring stability and increased specificity, but the combination in which they are used (i.e. chimeric, gapmer, and stereopure) are a niche titrating step as important to molecular precision as to toxicity.

Currently, one limitation to sequence-specific therapeutics might be that clinical trial pipelines eventually aiming at drug approval require every modification of a certain drug to undergo the full process of clinical testing, including phase 2 and 3 trials. Considering that about 130 amyloidogenic variants are known todate, it seems impossible (not only financially, but also in respect of low case numbers) to overcome these regulatory limitations for every single *TTR* mutation. Other than targeting the respective variant itself, however, allele-specific knock-down could also mean to target frequent polymorphisms located in *cis* with the mutated allele. This would require a more detailed genetic testing, including zygosity studies, but enable to optimize the development pipeline focusing on just a few frequent variations.

The CRISPR/Cas9 system, the subject of the 2020 Nobel Prize in chemistry, has garnered fame and support for its potential in permanently editing genomic DNA. This technique has the advantages 1) to reduce the application burden by permanent editing and 2) to specifically rewrite the causative DNA mutations. Clinical trials for Sickle cell disease and ß-Thalassemia, whose causal mutations are found in the ß-hemoglobin subunit gene *HBB*, have recently shown CRISPR/Cas9 to reduce production of a γ-globin repressor [61, 62]. For ATTRv amyloidosis, a Phase I open-label, multi-center study is currently underway in the United Kingdom to evaluate the safety, tolerability, and efficacy of NTLA-2001 sponsored by Intellia Therapeutics and Regeneron Pharmaceuticals [63]. Administered intravenously, with no ability to cross the blood–brain barrier, the CRISPR/Cas9 system travels predominantly to the liver in lipid nanoparticles. Using a single guide RNA, the system is designed to edit the TTR gene and completely disrupt its transcription by essentially creating a second knockout mutation. In the six eligible participants, none showed severe adverse events and all had a drastic decrease in serum TTR concentration, with those receiving the highest dose (0.3 mg/kg) experiencing an over 80% reduction 28 days after infusion [63]. Participants will be continually monitored and followed for 24 months after the initial infusion to determine sustained knockdown. While this relieves the primary burden of systemic disease, patients may continue to present with CNS and ocular symptoms as it will not affect TTR transcription other than hepatic.

Considering the amount of promising future treatment options for TTR, combination therapy may be the best tailored option using TTR stabilizers with the potential to cross the blood–brain barrier, intrathecally administered RNAi and ASOs, and genome editing strategies in a precision medicinal approach to eliminate the root cause.

### Are we creating a new phenotype? Ethical aspects

The availability of the aforementioned treatments for ATTRv amyloidosis is an undoubtedly positive development in terms of the possibility of increased lifespan and reduction of symptoms. Nevertheless, these treatments are not a cure for ATTRv amyloidosis, and raise myriad questions: What will happen when lifespans increase, but central amyloid production continues? Will we be able to mitigate neuropathy and cardiomyopathy, while treated patients have to face new issues including seizures, dementia, and blindness? What long-term impact do these treatments have on patients’ and their caregivers’ well-being and quality of life? Is this impact potentially greater than that of the initial phenotype? While the answers to some of these questions rely solely on clinical data that is not yet available, some raise specific ethical issues that we aim to illustrate in the following paragraphs.

- *Benefits vs. harms*

From an ethical standpoint, it is important to note that the risk of generating a new phenotype of a disease through treatment is not new, and has already been discussed in fields such as HIV, transplants [64], and, specifically in neurology, deep brain stimulation (DBS) for Parkinson’s Disease (PD) [65], and new treatments for amyotrophic lateral sclerosis (ALS) [66]. In all of these cases, the benefits of the treatment are evident and have been amply documented, as are the risks that come with an increased lifespan. Indeed, this discussion is likely to take place in an increasing number of clinical scenarios, as new treatment options increase patients’ lifespans, enabling known diseases to reach advanced stages of unknown severity [67]. In some cases, such as the one at hand, it is not only the severity of known symptoms, but the possibility of the emergence of new symptoms that is worrisome. From liver-transplanted ATTRv amyloidosis patients, we have learned that CNS and eye symptoms cannot be stopped if the choroid plexus and retina continue to produce mutant TTR. In the long-term, carriers of *TTR* mutations that are not specifically associated with predominant central involvement have survived long enough to eventually develop cerebral amyloid angiopathy or other CNS and eye manifestations. However, these risks and concerns must be weighed against the reality of ATTRv amyloidosis without treatment, and the evidence for currently available treatments. Thus, the main ethical questions we must ask are: Do the benefits of treatment outweigh the harms? Can these negative aspects of the new phenotype be treated with currently available agents, or is it foreseeable that they will be in the future? The currently approved medications cannot sufficiently overcome this problem. Should research focus shift to drugs that enforce TTR knockdown in the CNS and vitreous bodies as well? Or would that deprive these compartments from the protective roles of the TTR protein that might not play a relevant role in serum? Certain forms of treatment for ATTRv amyloidosis may come with a degree of iatrogenic harm, not only due to the emergence of a new phenotype, but also due to the currently limited perspective for successful treatment options that do not result in further harms resulting from the impairment of TTR functions. Our main focus now should be on the development of agents that can help prevent or treat the foreseeable symptoms of a possible new phenotype.

- *Informed consent and treatment plan*

In order to ethically implement treatment options that may give rise to a new phenotype of a disease, we must include all relevant information in the informed consent process, enabling an autonomous decision. An optimal clinical scenario would incorporate a written consent form that encompasses all relevant information, including: (a) a detailed description of the treatment and its many significant potential benefits, (b) the possibility of specific new symptoms, and c) a drafted future treatment plan. The aim of incorporating these aspects into informed consent discussions is not only to allow for careful consideration of both the potential benefits and consequences of treatment in order to decide whether to undergo the treatment at all (is the risk of blindness, dementia or epilepsy, among others, one that the patient is unwilling to accept when considering the benefit of the treatment?), but also to discuss what impact these risks would have on the patient’s perceived quality of life. Increasing patients’ lifespan while increasing their chances of blindness or dementia may mean that they will need care and may not be able to fulfill their expectations and values of a meaningful life or acceptable levels of well-being. It is also important to consider the risk of dementia and its effect on the capacity to consent to future treatments that may have positive effects on ATTRv amyloidosis. In order to safeguard patient autonomy, the discussion on whether to undergo treatment ought not be separated from the discussion of the patient’s wishes and values regarding their future treatment. Because of this, it is advisable to explicitly discuss drafting a future treatment plan, ideally in the form of advance directives, during the informed consent process.

Nevertheless, it is important that we underscore the proven benefits of the mentioned treatments during the process of informed consent: it is arguably as much a disservice to patient autonomy to insufficiently address risks as it is to overstate them or insufficiently address benefits. These scenarios could result, at worst, in a refusal of treatment due to a lack of hope in treatment, or, at best, to poor compliance.

- *Evidence-based decisions without evidence?*

Ethical clinical practice ideally ought to be based on data from well-designed randomized controlled clinical trials. This poses a challenge in rare diseases such as ATTRv amyloidosis, or treatment options that are considered “last resorts”, where patient numbers are lower and outcomes are less likely to be standardized. In addition, when effective treatment options are available, the ethical justification for placebo controlled trials where conditions for equipoise are not met becomes challenging at best. Due to the limited availability of data from clinical trials, authors have suggested the systematic collection of patient data [66] into rare disease registries with standardized treatment and outcome measures. The need for and potential benefits of these registries is highlighted in phenotype-modifying cases such as the one at hand, where a degree of uncertainty in certain aspects of treatment outcome is currently inevitable. As an example, the Transthyretin Amyloidosis Outome Survey (THAOS) has been systematically collecting demographic, clinical, and genetic data in more than 1400 ATTRv amyloidosis cases [68]. It does, however, not systematically assess the phenotypic changes under all available treatment options. This is a major limitation for the current question.

- *Stakeholder perspectives*

As mentioned above, liver-transplanted ATTRv amyloidosis patients present continued progression of CNS and eye symptoms if the choroid plexus and retina continue to produce mutant TTR. To the best of our knowledge, there are no published works focusing on the perspectives of patients and their caregivers on the development of new symptoms and their consequences for their well-being and quality of life. The same can be said on works exploring healthcare professionals’ experiences in this context and their attitudes towards treatment. Future practice and regulation in this area would greatly benefit from such input, and most importantly, could be extremely helpful in patients’ decision-making process.

## Conclusions

With the liver in the center of pathophysiology, ATTR amyloidosis has become a model disease for mechanism-based treatment approaches. Compared to other systemic or non-systemic diseases affecting the nervous system, the possibility to reduce the hepatic TTR production and thereby stop disease progression in almost all the other organs and tissues is a great advantage, given that various routes of administration are effective to target the liver. For the about 10% of extra-hepatic TTR production, there is, however, no good treatment strategy available to stop progression. It is conceivable that with longer survival, even *TTR* variants that have so far not primarily been associated with CNS and ocular disease manifestations might lead to clinically relevant amyloid angiopathy and vitreous opacities. That means that cerebral hemorrhage, dementia, and blindness might be the new phenotype of ATTRv amyloidosis in the long-term.

In the near future, it might be realistic to expect patients will receive RNAi combination therapies, one targeting primary TTR systemic production and the other targeting CNS specific TTR. Alternatively, physicians might be able to tailor either treatment strategy dependent on disease course and presenting symptoms.

To overcome the expected side effects of unspecific TTR knockdown in the CNS, an allele specific approach would be promising to selectively reduce the expression of the mutant, but not of the wildtype. Using the CRISPR gene editing technique, first steps are being made in that direction for ATTRv amyloidosis now, however, they have been, again, limited to hepatic TTR production so far.

In this review, we addressed and discussed two important questions that are both related to TTR function and dysfunction in the nervous system. First, we pointed out that the currently available treatment approaches do not target ATTR amyloid depositing in the eyes or CNS, which will eventually increase the impact of long-term “natural course” complications such as cerebral hemorrhage, dementia, and blindness with prolonged life-expectancies. Secondly, we emphasized that TTR might be of greater functional relevance in CSF than in serum, and that simple vitamin A substitution might not be sufficient to fully replace its neuroprotective effects. Taken together, central TTR knockdown might be helpful or even needed to stop CNS ATTRv amyloidosis, which is, without doubt, a dreadful and life-limiting condition. This can, on the other hand, potentially favor the development of dementia as well. TTR stabilization could be a compromise – at least in the function of bridging until disease progression eventually requires protein knockdown. This requires, however, a higher percentage of CSF availability.

Lastly, we considered the main ethical concerns raised:

- The generation of a new phenotype of ATTRv amyloidosis as a potential iatrogenic harm that must be weighed against the undoubted benefits of treatments
- The crucial importance of good practices in informed consent with detailed descriptions of both potential benefits and harms and the incorporation of a) a written informed consent form, and b) the discussion of advance directives into the process of informed consent
- The challenges of a paucity of data from randomized controlled trials and the importance of rare disease registries
- The need for stakeholder perspectives to inform future clinical practice and guidelines.

Great success has been made in the past decade turning ATTRv amyloidosis into a treatable disease and offering new perspectives to patients and families. To further improve these perspectives and to avoid new dreadful disease manifestations, many open questions will still have to be addressed in the future.

## Data Availability

Not applicable.

## Abbreviations

ALS: Amyotrophic lateral sclerosis
ASO: Antisense oligonucleotide
ATTRv amyloidosis: Hereditary transthyretin amyloidosis
ASGPR: Asialoglycoprotein receptor
CNS: Central nervous system
COMT: Catechol-*O*-methyltransferase
CRISPR: Clustered regularly interspaced short palindromic repeats
CSF: Cerebrospinal fluid
DBS: Deep brain stimulation
DNA: Desoxy-ribonucleic acid
EMA: European Medicines Agency
FDA: Food and Drug Administration
GalNac: N-Acetylgalactosamine
JAM: Junctional adhesion molecule
PD: Parkinson’s disease
RISC: RNA-induced silencing complex
RNA: Ribonucleic acid
siRNA: Small interfering RNA
SMA: Spinal muscular atrophy
THAOS: Transthyretin Amyloidosis Outome Survey
TTR: Transthyretin
UTR: Untranslated region
